# Evaluating Adherence to Antiretroviral Therapy Using Pharmacy Refill Records in a Rural Treatment Site in South Africa

**DOI:** 10.1155/2017/5456219

**Published:** 2017-01-31

**Authors:** George Gachara, Lufuno G. Mavhandu, Elizabeth T. Rogawski, Cecile Manhaeve, Pascal O. Bessong

**Affiliations:** ^1^HIV/AIDS & Global Health Research Programme, University of Venda, Thohoyandou, South Africa; ^2^Department of Medical Laboratory Sciences, Kenyatta University, Nairobi, Kenya; ^3^Department of Public Health Sciences, University of Virginia, Charlottesville, VA, USA; ^4^Division of Infectious Diseases and International Health, University of Virginia, Charlottesville, VA, USA; ^5^HIV/AIDS Prevention Group Wellness and Health Clinic, Bela Bela, South Africa

## Abstract

Optimal adherence to combination antiretroviral therapy (cART) is critical to maintain virologic suppression, thereby ensuring the global success of HIV treatment. We evaluated adherence to cART using pharmacy refill records and determined the adherence threshold resulting in >90% virologic suppression in a community run treatment site in South Africa. Additionally, we analysed factors associated with adherence using univariable and multivariable logistic regression models. Logistic regression was also performed to determine the relationship between adherence and virologic suppression and the adherence threshold resulting in <10% virologic failure. The overall median (interquartile range) adherence was 95% (88.6–98.4%). Out of the study participants, 210/401 (52.4%) had optimal (≥95%) adherence while only 37/401 (9.2%) had poor (≤80%) adherence. The majority (90.5%) of patients with optimal adherence had virologic suppression. Having TB at registration into care was found to be negatively associated with adherence (adjusted odds ratio [AOR], 0.382; *p* ≤ .05). Compared to nonadherent individuals, optimally adherent participants were more likely to achieve virologic suppression (OR 2.92; 95% CI: 1.63–5.22). Only adherence rates above 95% were observed to lead to <10% virologic failure. cART adherence measured by pharmacy refill records could serve as a useful predictor of virologic failure; adherence rates >95% are needed to maintain optimal virologic suppression.

## 1. Introduction

Strict adherence to combination antiretroviral therapy (cART) is usually critical to a sustained HIV suppression, as well as decreased risk of HIV transmission, reduced risk of drug resistance, improved overall health, quality of life, and survival. As the world aims to achieve zero new HIV infections, there is renewed focus to maintain long-term adherence, especially given that therapeutic options beyond second-line are more expensive and often nonexistent in most countries [1]. The critical role of adherence to antiretroviral therapy to achieve successful treatment of HIV infection has prompted research into adherence and increased clinician interest in an attempt to address adherence issues in the context of ongoing patient care [2].

A number of strategies are available to measure adherence to cART. Each of these methods is associated with certain strengths and drawbacks. Consequently, there is no gold standard measure of adherence. However, recent studies have suggested that pharmacy refill records are a more accurate measure of adherence than patient self-report and clinic-based pill counts and that they correlate well with HIV outcomes in resource-limited settings [3].

Following introduction of universal cART access in public health facilities in South Africa in 2004, instances of ARV resistance and treatment failure have been observed. The Limpopo province, in northern South Africa, has a high level of poverty with majority of its population living in rural areas and with a HIV prevalence of up to 30% in antenatal mothers. A previous study suggested a moderately high level of resistant viruses among individuals visiting a voluntary counseling and testing centre in the province [4]. It has therefore been hypothesized that the increasing number of individuals on cART in this region will lead to higher levels of virologic failure resulting in a higher prevalence of transmitted and acquired drug resistance. Quantifying and monitoring adherence to cART is one potentially useful and low-cost method of identifying patients at high risk for virologic failure in resource-limited settings. There is a dearth of information on adherence rates in the Limpopo Province of South Africa. This study therefore sought to review the relationship between adherence, measured using pharmacy records, and viral outcomes in one treatment facility in the region. The aim was to identify a threshold adherence rate that could be used in real-time to intervene on patients at risk of virologic failure.

## 2. Methods

### 2.1. Study Site and Design

This was a retrospective assessment undertaken at the HIV/AIDS Prevention Group (HAPG) Wellness Clinic in Bela Bela, Waterberg District in Limpopo province, South Africa. The HAPG clinic has been providing medical care to HIV infected individuals free of cost, including medicines, consultations, CD4 cell count enumeration, and HIV viral load since 2001. Included in the study were all patients who initiated cART at the clinic between December 2004 and April 2015, who were on cART for at least six months and had at least two CD4 and viral load test results. The available patient demographics, pharmacy refill, clinical, viral load, and CD4 cell count data were obtained from clinic records and used for subsequent analyses. The study was approved by the Research Ethics Committee of the University of Venda, South Africa (SMNS/15/MBY/23/0710).

### 2.2. Patient Evaluation and Care

Patients enrolling for care at this clinic, approximately 30 per day, were first counseled and then tested for HIV. Individuals testing positive for HIV are assessed for ART eligibility according to the national guidelines [5–7]. Those found eligible were initiated on treatment after undergoing integrated individual adherence support counseling. Patients were then provided with a monthly supply of drugs, projected monthly pharmacy refill dates, and the date for the next appointment. During these subsequent monthly visits, adherence counseling was continued until one year after treatment initiation when a “certificate of knowledge on adherence” was awarded. Clinic-based pill counts were not conducted during these visits. Patients not eligible for ART were registered into pre-ART care involving biannual clinical and immunological assessments for ART eligibility as per the national guidelines.

During the study period considered, patients were on a number of treatment regimens. Between 2004 and 2010, all adults were started on Stavudine (d4T), Lamivudine (3TC), and Efavirenz (EFV) or Nevirapine (NVP), with Ritonavir booted Lopinavir (RTV/r) for children. Second-line therapy comprised Zidovudine (AZT), Didanosine (ddI), and LPV/r. In 2010, the first-line regimen used included Tenofovir (TDF), Emtricitabine (FTC), or 3TC and EFV/NVP. d4T + 3TC + EFV and AZT + 3TC + EFV/NVP were used as alternative regimens. The second-line regimen was TDF + 3TC/FTC + LPV/r for those failing a d4T or AZT-based first line regimen and AZT + 3TC + LPV/r for those failing a TDF based first-line regimen. All patients initiating therapy from 2013 were put on TDF + FTC/3TC + EFV as a fixed dose combination (FDC) with TDF + FTC/3TC + NVP, AZT + 3TC + EFV/NVP, d4T + 3TC + EFV/NVP, and Abacavir (ABC) + 3TC + EFV/NVP as alternative regimens. For second-line therapy, AZT + 3TC + LPV/r or TDF + 3TC/FTC + LPV/r were administered. In 2015, the 2013 recommendations were largely retained except that ABC + 3TC + LPV/r was also used as an alternative second-line regimen.

### 2.3. Adherence Measurement

In this study, adherence to cART was defined by the regular monthly collection of prescribed cART from the clinic's pharmacy. Each refill period was identified as the interval between the last pharmacy visit date and the scheduled refill date. Refill adherence was 100% if all pills during the scheduled refill period were picked up on time. Refill percent values above 100% for patients who refilled earlier than scheduled were rounded to 100%. Refill adherence was calculated based on the cumulative sum of days that a patient was late for cART pick-up appointments, divided by the total number of days over all such periods in the study, resulting in the percentage of time the patient was without medication over the whole study period. Patients were categorized as optimally adherent (>95%), suboptimally adherent (80–94%), and poorly adherent (<80%), as previously described [8]. Data pertinent to this study was sourced from the clinic records. Virologic failure was defined as a plasma HIV RNA level >400 copies/*μ*l at the last viral load determination.

### 2.4. Statistical Analyses

Statistical analysis was performed using SPSS version 20 (IBM, Chicago, IL, USA). We used descriptive statistics to present baseline patient characteristics and univariable logistic regression to determine presence of associations between potential predictors and the outcome variable (adherence). All independent variables that were statistically associated (<0.1) with the outcome variable in univariate analyses were included in the final model. A multivariable logistic regression model was then constructed for adherence to identify the predictors at a 5% level of significance. Virologic failure rates were estimated for patients within categorized levels of adherence and logistic regression was used to estimate the overall relationship between virologic failure and varying levels of adherence.

## 3. Results

### 3.1. Cohort Description

A total of 401 out of 847 records met the study criteria and were reviewed. The mean age was 36.5 years (interquartile range [IQR] 30–45). The majority (246, 61.3%) were females and among those with education qualifications recorded, 127 (63.5%) had secondary school or higher education. Most of the patients (211, 52.7%) initiated treatment at the clinic with WHO stage 3 or 4 AIDS disease. The mean initial CD4 cell at cART initiation was 273 cells/*μ*l (IQR, 115–350) and mean viral load was 304,141 copies/*μ*l (IQR, 13,626–273,638). The mean duration under cART among the study participants was 2.2 years (IQR 0.7–2.6). Baseline characteristics of the study population are shown in Table 1.

### 3.2. Rates of Adherence

Among the 410 patients, 210 (52.4%) had optimal adherence (≥95% pill refills collected on time), 154 (38.4%) had suboptimal adherence (80–94%), and 37 (9.2%) had poor adherence (<80%). The overall median cART adherence was 95% (IQR; 88.6–98.4%). These results are shown in Table 2.

### 3.3. Factors Associated with Adherence to cART

In univariable analyses, females were found to be more adherent than males though the estimate was not statistically significant (OR: 1.4, 95% CI: 0.94–2.11). Older people and those initiating cART with WHO stage 4 disease were also more likely to be adherent though this relationship was also not statistically significant (OR: 1.29, 95% CI: 0.78–2.15 and OR: 2.00, 95% CI: 0.77–5.20, resp.). Patients diagnosed with tuberculosis (TB) at registration into care were less likely to be adherent (OR: 0.38, 95% CI: 0.16–0.90). In multivariable analysis, only TB at registration was significantly associated with adherence (OR: 0.38, 95% CI: 0.16–0.92). Patients without TB at registration were 2.6 times more likely to be adherent (Table 3).

### 3.4. Adherence and Virologic Suppression

**Figure 1:**
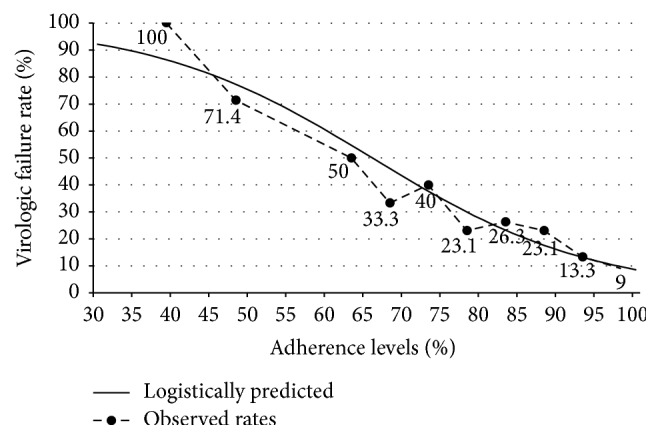
Observed and model-predicted virologic failure rates by adherence levels. The observed rates were calculated at specific adherence level categories, while the predicted rates were estimated across the continuous range of adherence.

Overall, we found that 84.5% (*n* = 339) patients achieved virologic suppression at their last viral load determination. Patients with optimal adherence were almost three times as likely to be virologically suppressed compared to the nonoptimally adherent patients (OR 2.92; 95% CI: 1.63–5.22). As expected, a high level of adherence corresponded to low virologic failure. Virologic failure was inversely related to the adherence rates and the logistic regression predicted virologic failure rates generally fell within the observed rates. Adherence levels of 90–94% resulted in 10–20% virologic failure while only 95–100% adherence levels resulted in <10% virologic failure (Figure 1).

## 4. Discussion

In this study, we found an overall median adherence of 95%, such that slightly more than half (52.4%) of the study population had optimal adherence (>95% drug refills on time). This level of adherence is similar to the median adherence of 94% from Nigeria [7] and 95% in Pretoria, South Africa [8]. The proportion with optimal adherence is marginally lower than the 53.4%–99.8% optimal adherence rates described in various studies across Africa [9] and also lower than the 62.5% observed in another rural health centre in South Africa [10]. Among those with optimal adherence to drug-refill schedules (at least 95% on time), 91% (191/210) of the patients had virologic suppression. In resource-limited settings, as suggested recently, this has clinical implications as systematic monitoring of pharmacy refill adherence could be used as a predictor of higher chances of virologic failure [7]. According to the WHO, adherence to antiretroviral agents varies between 37% and 83% [11]. However, it should be noted that most studies have reported adherence rates over shorter durations than the ones covered in this study. There often exist challenges in maintaining the often impressive early performance since adherence may wane over time.

Among the independent factors analysed in this study, multivariate analysis found only TB diagnosis at registration into care to be associated with adherence. Patients diagnosed with TB during registration into care were found to be more nonadherent compared to those who tested TB negative. It is possible that this may be due to TB-immune reconstitution inflammatory syndrome (TB-IRIS), a worsening or recurring of preexisting tuberculous lesions in patients on effective antituberculosis treatment which often occurs after cART initiation. It should be noted that majority of patients are often initiated into cART immediately after registration into care due to late presentation at the facility. Previous studies have reported that up to one-third of patients with HIV/TB coinfection who begin cART are at risk of TB-IRIS [9]. The resultant clinical manifestations may lead to depression, negative feelings, and loss of hope which can reduce the motivation of the patients to adhere [10]. Since having a diagnosis of TB before or after registration into care was not associated with adherence, we therefore suggest that the collateral effects of initiating cART in these patients compromise their social life negatively affecting adherence. It is also possible that the resulting pill burden from HIV/TB medication may also negatively affect adherence as has been shown previously [11]. We recommend repeated intensified adherence counseling in this group.

Compared to nonadherent individuals, adherent participants were more than two and a half times as likely to achieve virologic suppression. This is expected since holding all other factors constant, viral load is significantly related to the plasma concentration of the drug. Higher levels of virologic failure were associated with lower levels of adherence, with failure rates rising steeply below the 70–74% adherence range. While 90–94% adherence rates were found to lead to virologic failure rates <20%, only the >95% adherence range met the threshold for an outcome of <10% virologic failure in the current study. Some studies have suggested that 70–80% adherence meets the threshold for an outcome of <20% virologic failure rate. This however has only been accomplished using the newer, more potent cART regimens including boosted protease and integrase inhibitors [12] which were not in use at this clinic. Adherence >95% is generally considered the gold standard of adherence necessary to maintain virologic suppression in majority of individuals [13].

### 4.1. Study Limitations

The findings in the current study should be understood in the context of several limitations. First, the cross sectional design of the study does not reflect the dynamic nature of adherence which can vary over time. Secondly, the retrospective nature of the study only allows for the examination of factors that have been recorded and not others which have been associated with adherence in other studies such as income and adverse reactions. Lastly, the study included only patients who remained in care at the clinic and had outcome measurements available. This may overestimate adherence since it is likely that patients who were lost to care and could not be included would have been much less adherent and had worse outcomes.

### 4.2. Conclusions

In conclusion, this study finds that adherence to cART measured by pharmacy refill records could serve as a useful predictor of virologic failure in this setting. TB diagnosis during registration into care negatively affected adherence. Enhanced adherence support for TB patients entering into HIV care is recommended.

## Figures and Tables

**Table 1:** Baseline characteristics of the study participants.

Characteristic	Number of patients (%)
Age, years	
≤35	175 (43.6)
36–45	135 (33.7)
≥46	91 (22.7)
Age, mean years (IQR)	36.5 (30–45)
Female (%)	246 (61.3)
Duration under cART, mean years (IQR)	2.2 (0.7–2.6)
WHO stage (%)	
I or II	178 (44.4)
III or IV	211 (52.7)
Missing	12 (3.0)
Mean initial CD4 (cells/*µ*l) [IQR]	273 (115–350)
Initial CD4 strata (%)	
≤100 cells/*µ*l	92 (22.9)
101–200 cells/*µ*l	83 (20.7)
201–350 cells/*µ*l	125 (31.2)
≥350 cells/*µ*l	99 (24.7)
Missing	2 (0.5)
Mean initial viral load (copies/ml) [IQR]	304,141 (13626–273638)
Initial viral load strata (%)	
≤100000 copies/ml	206 (51.4)
≥100000 copies/ml	175 (43.6)
Missing	20 (5.0)

**Table 2:** Adherence to cART among the study participants.

Patient group by treatment adherence	Number (%) of patients (*n* = 401)
Above 95%	210 (52.4)
90% to 94%	83 (20.7)
85% to 89%	52 (13.0)
80% to 84%	19 (4.7)
75% to 79%	13 (3.2)
Below 75%	24 (6.0)
*Total*	*401 (100)*

*Median adherence 95%* and * IQR 88.5–98.4*	

**Table 3:** Association between cART adherence and selected sociodemographic and clinical risk factors.

Variable	Number (%) of patients		Unadjusted OR (95% CI)	Adjusted OR (95% CI)
	Adherent	Nonadherent		
Sex				
Male	73 (34.8)	82 (42.9)	Referent	
Female	137 (65.2)	109 (57.1)	1.412 (0.943–2.113)^c^	1.398 (0.924–2.113)
Age group (years)				
≤35	87 (41.4)	88 (46.1)	Referent	
36–45	72 (34.3)	63 (33.0)	1.156 (0.737–1.812)	
≥45	51 (24.3)	40 (20.9)	1.290 (0.775–2.146)	
Duration under cART (days)				
≤365	93 (44.3)	70 (36.6)	Referent	
≥365	117 (55.7)	121 (63.4)	0.728 (0.487–1.087)	
WHO stage^a^				
Stage 1	13 (6.2)	13 (7.2)	Referent	
Stage 2	81 (38.8)	71 (39.4)	1.141 (0.496–2.622)	
Stage 3	79 (37.8)	78 (43.3)	1.013 (0.442–2.323)	
Stage 4	36 (17.2)	18 (10.0)	2.000 (0.770–5.195)	
TB prior to registration into care				
TB positive	16 (7.6)	15 (7.9)	Referent	
TB negative	194 (92.4)	176 (92.1)	0.968 (0.465–2.015)	
TB at registration into care				
TB positive	8 (3.8)	18 (9.4)	Referent	
TB negative	202 (96.2)	173 (90.6)	0.381 (0.162–0.897)^b^	0.382 (0.159–0.916)^b^
TB after registration into care				
TB positive	28 (13.3)	22 (11.5)	Referent	
TB negative	182 (86.7)	169 (88.5)	0.846 (0.466–1.536)	
Starting cART regimen				
FDC	115 (54.8)	99 (51.8)	Referent	
Other	95 (45.2)	92 (48.2)	0.889 (0.600–1.317)	
Educational level^a^				
Primary school/no education	36 (35.3)	37 (37.8)	Referent	
Secondary school or higher	66 (64.7)	61 (62.2)	1.112 (0.625–1.978)	

^a^Not all patients had this variable recorded.

^b^ *p* ≤ .05 and  ^c^*p* ≤ .10.
